# Infectious bursal disease virus inoculation infection modifies *Campylobacter jejuni*–host interaction in broilers

**DOI:** 10.1186/s13099-018-0241-1

**Published:** 2018-03-30

**Authors:** Li Li, Colin Pielsticker, Zifeng Han, Tereza Kubasová, Ivan Rychlik, Bernd Kaspers, Silke Rautenschlein

**Affiliations:** 1 Clinic for Poultry, University of Veterinary Medicine Hannover, Bünteweg 17, 30559 Hannover, Germany; 2 Veterinary Research Institute, Hudcova 70, 621 00 Brno, Czech Republic; 3 Department for Veterinary Sciences, Faculty of Veterinary Medicine, Ludwig-Maximilians-Universität München, Veterinastr. 13, 80539 Munich, Germany

**Keywords:** *Campylobacter jejuni*, Infectious bursal disease virus, Immunosuppression, Immune response, Gut microbiota composition

## Abstract

**Background:**

*Campylobacter jejuni* is considered as a chicken commensal. The gut microbiota and the immune status of the host may affect its colonization. Infectious bursal disease virus (IBDV) is an immunosuppressive virus of chickens, which allows secondary pathogens to invade or exacerbates their pathogenesis. To investigate the effect of IBDV-induced immunosuppression on the pathogenesis of *C. jejuni*, broiler chickens were inoculated with a very virulent (vv) strain of IBDV at 14 days post hatch followed by *C. jejuni* inoculation at 7 (Experiment A) or 9 (Experiment B) days post virus (IBDV) inoculation.

**Results:**

vvIBDV-infection led to a depression in caecal lamina propria B lymphocytes and the anti-*C. jejuni*-antibody response starting at 14 days post *C. jejuni* inoculation (pbi). The C. *jejuni*-colonization pattern was comparable between mono-inoculated groups of both experiments, but it varied for vvIBDV + *C. jejuni* co-inoculated groups. In Experiment A significant higher numbers of colony forming units (CFU) of *C. jejuni* were detected in the caecum of co-inoculated birds compared to *C. jejuni*-mono-inoculated birds in the early phase after *C. jejuni*-inoculation. In Experiment B the clearance phase was affected in the co-inoculated group with significantly higher CFU at 21 days pbi compared to the mono-inoculated group (*P* < 0.05). No major differences were seen in numbers local lamina propria T lymphocyte populations between *C. jejuni*-inoculated groups with or without vvIBDV-infection. Interestingly, both pathogens affected the microbiota composition. The consequences of these microflora changes for the host have to be elucidated further.

**Conclusion:**

Our data suggests that the timing between viral and bacterial infection might affect the outcome of *C. jejuni* colonization differently. Our results confirm previous studies that anti-*Campylobacter*-antibodies may specifically be important for the clearance phase of the bacteria. Therefore, as vvIBDV is widely distributed in the field, it may have a significant impact on the colonization and shedding rate of *C. jejuni* in commercial poultry flocks. Subsequently, successful IBDV-control strategies may indirectly also benefit the gut-health of chickens.

**Electronic supplementary material:**

The online version of this article (10.1186/s13099-018-0241-1) contains supplementary material, which is available to authorized users.

## Background

*Campylobacter jejuni* is the leading cause of bacterial food-borne gastroenteritis in humans in industrial countries. Poultry is considered as the main reservoir for *C. jejuni*, with high bacterial loads in the gastrointestinal tract. *C. jejuni* has been regarded as a commensal for chickens [1]. However, recent studies have reported that *C. jejuni* may induce a mild inflammatory response and affects the gut morphology in colonized chickens [2, 3]. It is therefore suggested that *C. jejuni* may have a substantial impact on the chicken’s health and welfare [2].

Different risk factors may affect the colonization pattern of *C. jejuni* in chickens including strain to strain variation, the inoculation dose, host genotype, management as well as water and feed composition [3, 4]. Poor gut health and compromised immunity are considered to negatively influence the chicken’s health [2].

Different pathogens may modify the functionality of the immune system [4]. Infectious bursal disease virus (IBDV) is one of the most important immunosuppressive viruses affecting the chickens worldwide [5]. Both humoral and cell-mediated immune responses (CMI) are affected. IBDV-infected birds show systemic as well as local depletion of B cells, infiltration of T cell subsets in the bursa of Fabricius (BF) and modulation of innate immune parameters [5–7]. IBDV infection leads to a strong upregulation of proinflammatory mediators and cytokines, a so called ‘cytokine storm’, in the acute phase and even cause death during this period [6, 7]. Surviving chickens may suffer from permanent immunosuppression when they were infected early in life [5, 8]. Immunosuppressed chickens are more susceptible to secondary infections, which was experimentally demonstrated after IBDV-co-inoculation with *Salmonella typhimurium* (ST) and *Escherichia coli* [9, 10]. Inoculation of specific-pathogen-free (SPF) birds with an IBDV Del-E strain led to an increase in ST shedding, and anti-ST immune reactions were dramatically impaired in co-infected birds [10]. Increased *C. jejuni* colonization and shedding was demonstrated in chickens co-inoculated with a Del-E strain of IBDV and *C. jejuni* [11]. Another study showed that vaccination of chickens with an intermediate IBDV strain led to lesion development in the gut and liver when birds were also inoculated with *C. jejuni* in comparison to *C. jejuni* mono-inoculated birds [12]. However, the mechanisms leading to an exacerbation of *C. jejuni* colonization are not fully clear. We speculate that IBDV may modify the local *C. jejuni* colonization pattern. It may compromise the induction of *C. jejuni*-specific humoral immunity and may have possibly other direct or indirect effects on gut immunity [13] and microbiota composition. It was suggested that maternal antibodies may protect against *C. jejuni* colonization [14]. However, the role of humoral immunity in *Campylobacter* control has been discussed controversially [15]. Recent studies with chemically B cell-compromised chickens indicated that humoral immunity may be important in the clearance of *C. jejuni* from the small intestine [16].

In the present study, commercial broilers were inoculated with a very virulent (vv) IBDV strain at 2 weeks post hatch, when maternally derived anti-IBDV antibodies were below the break-through level of the virus. IBDV-induced suppression of circulation B cells was confirmed starting at 3 days post virus (IBDV) inoculation (pvi) in both experiments lasting up to 9 days pvi. Subgroups of vvIBDV-inoculated and virus-free birds were subsequently orally inoculated with *C. jejuni* at two different time points expecting birds to be at different stages of IBDV-pathogenesis and induced immunosuppression: In experiment (Exp.) A, birds were *C. jejuni*-inoculated at seven and in Exp. B at 9 days pvi. Lesion development, replication of pathogens as well as gut associated immune parameters and microbiota composition were determined.

## Methods

### Inocula of vvIBDV and *C. jejuni*

The vvIBDV strain 89163/7.3 was kindly provided by N. Eterradossi, AFSSA, Ploufragan, France [17]. vvIBDV propagation was described before [17]. A challenge dosage of 10^3^ egg infectious dose (EID)_50_/bird via eye drop was used.

The *C. jejuni* strain Lior 6 was isolated from a chicken at the Clinic for Poultry, University of Veterinary Medicine Hannover, Germany [3]. The preparation and quantification of the inoculum was described previously [3]. Briefly, the bacteria, which were stored at − 80 °C in skim milk, were thawed and incubated for 48 h at 38 °C on Charcoal Cefoperazone Deoxycholate Agar (CCDA, Oxoid, Basingstoke, England) under microaerobic conditions. After an additional 48 h of incubation, one colony of *C. jejuni* was transferred into three ml Standard-I-Bouillon (Merck, Darmstadt, Germany) and incubated for another 48 h under microaerobic conditions at 38 °C. The inoculation dose in both experiments was adjusted to 10^6^ colony forming units (CFU) per chicken in 1 ml of sterile PBS and was applied via crop inoculation. The exact bacterial concentration was obtained by plating the inocula on CCDA plates in tenfold dilution series [3]. After microaerobic incubation at 38 °C for 48 h the colonies were counted to calculate the actual CFU/inoculum [18]. The CFU numbers of the inoculum were confirmed to be 10^6.57^ and 10^6.7^ CFU/ml in Exp. A and B, respectively.

### Cell isolation and flow cytometric analysis of circulating Bu1 + lymphocytes

Full blood samples with the addition of EDTA were collected (0.5 ml/bird, n = 10/group) and diluted in the same amount of PBS. A gradient centrifugation (Biocoll, 1.09 g/ml; Biochrom AG, Berlin) was performed for the isolation of lymphocytes according to manufacturer’s instructions [19]. For one-color staining, 2 × 10^5^ cells/sample were stained with anti-chicken Bu1 + monoclonal antibodies labeled with fluorescein isothiocyanate (FITC) at a concentration of 0.05 µg/ml (Southern Biotech, provided by Biozol, Eching, Germany) on ice for 30 min in the dark. After three washing steps with PBS containing 1% fetal bovine serum (FBS, Sigma, USA), the cells were acquired by the BD FACSCanto II (Becton–Dickinson, Heidelberg, Germany). Samples were analyzed with the BD FACS DIVA software (Tree Star Inc., OR, USA). Total leukocytes were analyzed by forward and side ward scatter analysis, dead cells were excluded by size, a lymphocyte gate was set, and the percentage of Bu1 positive B cells was determined within this gate.

### Histology

Samples of the bursa of Fabricius (BF) and the middle region of the caecum were collected, fixed in 4% (w/v) phosphate-buffered formalin, embedded in paraffin, sectioned (2 µm) and further processed for histological evaluation following standard procedures. The histopathological lesions of the BF were determined via light microscopy [20]. Data are presented as % follicles, which show more than 50% lymphoid cell depletion. Caecum lesions including loss of epithelial integrity, edema, infiltration of plasma cells as well as heterophils were also evaluated [2].

### IBDV-detection by immunohistochemistry

Bursae were collected, fixed in 4% phosphate-buffered formalin, sectioned and processed as previously described [18]. Sections of 2 µm were stained using the Universal Vectorstain^®^ Kit (Vector Laboratories, Burlingame, CA, USA). A polyclonal rabbit anti-IBDV serum was used for IBDV-antigen detection [6]. The group means of the number of IBDV-antigen positive cells per field at a magnification of 200× were calculated based on the average number of positive cells in 10 randomly selected microscopic fields for each bird per group.

### Detection of immune cells by immunohistochemistry

The BF and the middle region of the caecum were snap-frozen in liquid nitrogen, and sections of 4 µm were prepared. The following mouse-anti-chicken primary unlabeled antibodies were used: anti-CD4 (clone CT-4), anti-CD8β (clone EP-42) and anti-Bu1 (21-1A1) all at a concentration of 0.05 µg/ml (Southern Biotech, provided by Biozol, Eching, Germany). The secondary anti-mouse IgG biotinylated antibodies were used subsequently and the enzyme-linked ABC complex was visualized by the reaction with 3.3′-diaminobenzidine (DAB) chromagen substrate and hydrogen peroxide (DAB peroxidase substrate Kit, Vector Laboratories Inc.) [3]. Sections were examined by light microscopy. The T-lymphocyte populations in the BF were evaluated by counting the number of stained cells at a magnification of 200× in five randomly selected microscopic fields per bird [6]. The B and T lymphocyte populations in the lamina propria (LP) of the caecum were counted in three crypts/field of five randomly selected fields for each bird at a magnification of 200× [3].

### Quantification of the *C. jejuni* load in caecal content and reisolation from other organs

*Campylobacter jejuni* quantification was done according to a previous study [18]. From each *C. jejuni*-inoculated chicken, one whole caecum was collected. Around 0.5 g content was tenfold serially diluted with phosphate buffered saline (PBS). 100 µl of the diluted samples were spread on CCDA plates in duplicates. In addition 1 g of liver was collected from each bird, homogenized with 3.5 ml distilled PBS and spread on CCDA plates in duplicates. For qualitative *C. jejuni* reisolation from other tissues, swab samples of spleen, ileum and BF were collected and plated directly on CCDA plates. The plates were incubated under microaerophilic condition at 38 °C for 48 h. The calculation of CFU/g was described previously [3, 18].

### RNA extraction and real-time quantitative RT-PCR (qRT-PCR) for the detection of cytokine expression

Total RNA was isolated from the BF using 1 ml Trifast^®^-GOLD reagent (PeqLab, Biotechnologie GmbH, Erlangen, Germany) as previously described [3]. RNA quality and concentrations were determined using the NanoDrop ND-1000 (PeqLab, Biotechnologie GmbH). Probes and primers for the detection of interferon (IFN)-γ, interleukin (IL-6), and the IL-8 were described before [18, 21]. Real-time quantitative RT-PCR was performed with 20 µl reaction mix using the Qscript™ XLT One-Step RT-qPCR ToughMix Kit (Applied Biosystems, AMBio.n, USA). Amplification and analysis was done with the Mx3005P™ thermal cycle system and the Mx3005P™ Q PCR Software (Stratagene, Agilent Technologies Company, USA), respectively. The following cycle profile was applied: one cycle at 50 °C for 10 min, at 95 °C for 1 min, followed by 40 cycles at 95 °C for 10 s and 60 °C for 1 min. The data were normalized by using the house-keeping gene 28S [18], which was expressed in a comparable manner in all samples of infected and non-infected groups. Results are presented as 40-Ct. All the samples were tested in duplicates.

### ELISAs for anti-IBDV and anti-*C. jejuni* antibody detection

The commercially available enzyme-linked immunosorbent assay (ELISA) ProFlok IBDV plus antibody test kit (Synbiotics Co., Kansas City, Mo.) was used to detect circulating anti-IBDV-antibodies [6]. An inhouse biotin-streptavidin-based ELISA was applied for the detection of anti-*C. jejuni* IgG-type specific antibodies as previously described [22]. Anti-IBDV-antibody titers are presented as mean titer/group ± standard deviation (SD). Average *C. jejuni*-specific antibodies levels are expressed as average OD-values ± SD.

### Gut microbiota composition

Following the homogenization of caecal content using zirconia silica beads in a MagNALyzer (Roche Diagnostics, Basel, Switzerland), DNA was isolated with the QIAamp DNA Stool Mini kit according to the manufacturer’s instructions (Qiagen, Hilden, Germany). The DNA quality and concentration were measured spectrophotometrically, and the sequencing of the V3/V4 variable region of the 16S rRNA genes was performed as described before [23]. DNA samples were diluted to 5 µg/µl and used as a template with forward primer 5′CGTATCGCCTCCCTCGCGCCATCAG–MID–GGAGGCAGCAGTRRGGAAT3′, and reverse primer 5′ CTATGCGCCTTGCCAGCCCGCTCAG–MID–CTACCRGGGTATCTAATCC3′. The MIDs represent different sequences (5, 6, 7, 9 or 12 bp in length) which were designed to differentiate samples into groups. The underlined sequences were required for amplification of overlapping the V3/V4 region of 16S rRNA genes. The KAPA Taq HotStart PCR Kit was used for PCR amplification and cleanup following manufacturer’s instructions (Kapa Biosystems, Boston, MA, USA). The following cycle profile was applied: hot start at 95 °C for 15 min and 95 °C for 1 min, followed by 30 repeat cycles consisting of incubation at 94 °C for 40 s, 55 °C for 55 s and 72 °C for 1 min. Amplification products were pooled and indexed with a Nextera XT index kit and sequenced using the MiSeq Reagent Kit v3 and MiSeq apparatus according to the manufacturer´s instruction (Illumina). QIIME software was used for processing the sequencing data [23]. The sequences which are presented were classified by RDP Seqmatch with OTUs (operational taxonomic units), and the principal coordinate analysis (PCoA) was used for data visualization.

### Animals and experimental procedure

Two experiments were conducted with four groups each: the non-inoculated control (control-group), the vvIBDV mono-inoculated (vvIBDV-group) and the *C. jejuni* mono-inoculated group (*C. jejuni*-group), as well as the vvIBDV + *C. jejuni* co-inoculated group (co-inoculation-group).

### Animals

One-day-old commercial broiler chicks (Ross 308) were purchased from the Hatchery Weser-Ems (BWE), Visbek, Rechterfeld, Germany. All broilers were raised in one room at the Clinic for Poultry under isolation conditions on wood shavings until the age of 14 days. Afterwards, birds were randomly assigned to different groups and moved to different isolation rooms or Horsfall-Bauer-type isolators. Birds of all the groups received commercial broiler feed and water from the same source throughout the experiments, which were provided ad libitum. The chicks did not receive any vaccination. Experiments were conducted according to the regulations for animal welfare of Lower Saxony and were approved by the Lower Saxony State Office for Customer Protection and Food Safety (LAVES: 33.12-42505-04-13/1215).

### Experiment A (Exp. A)

Seventy-two one-day-old commercial broiler chicks (Ross 308) were used in Exp. A. Serum samples (n = 15) were randomly collected at seven and 14 days post hatch and anti-IBDV antibodies were determined by ELISA. Anti-IBDV ELISA-titers ranged from log_10_ of 1.7–3.7 for individual birds, which suggests low maternal anti-IBDV antibody titers according to the information provided by the ELISA kit manufacturer (ProFLOK PLUS-ELISA kit, Zoetis, US) allowing early vvIBDV-infection around day 14 post hatch [24, 25]. At 14 days post hatch broilers were distributed randomly into two groups. One group was inoculated with 10^3^ EID_50_/bird of vvIBDV by eye-drop route and the other group (control) received only the diluent PBS. At 3, 5 and 7 day pvi, blood samples were collected (n = 8/group). Peripheral blood leukocytes (PBL) were isolated and analyzed by flow cytometric analysis for the percentage of circulating Bu1-positive B cells. At the same time, cloacal swabs were collected to confirm that birds were *C. jejuni*-free. At 7 days pvi, when circulating B cell numbers were significantly decreased in the vvIBDV mono-inoculated group compared to the virus-free birds (*P* < 0.05), groups were divided into two subgroups (18 birds/subgroup) and orally inoculated with 10^6^ CFU of *C. jejuni* or PBS. Clinical signs were monitored daily throughout the experiment. Six birds from each group were sacrificed at three, seven and 14 days pbi and pathological lesions were determined. BF, liver and spleen were collected to determine organ to body weight ratios. Serum samples were used for the detection of anti-IBDV and *C. jejuni* antibodies by ELISA. Samples of the BF and caecum were taken for immunohistochemical detection of different immune cell populations as well as IBDV-antigen positive cells. Since the BF is the target organ for IBDV replication, the BF was also collected for the detection of cytokine mRNA expression by qRT-PCR at three, seven and 14 days pbi. The caecal content was evaluated for microbiota composition by Illumina sequencing at 7 days pbi.

### Experiment B (Exp. B)

Ninety-six one-day-old broiler chickens were used in Exp. B. The experimental setup of Exp. B was comparable to Exp. A with the exception that *C. jejuni* inoculation took place at 9 days pvi. An additional flow cytometric analysis of circulating B cells was conducted with blood samples in Exp. B. Six birds from each group were sacrificed for necropsy not only at three, seven and 14 days but also at 21 days post bacteria inoculation, and collected samples were processed as indicated for Exp. A. Unlike in Exp. A cytokine mRNA expression levels in the BF were only determined at 3 days pbi.

### Statistical analysis

One bird was chosen as the unit for analysis. Statistical analysis was performed with the Statistix version 9.0 (Analytical software, Thallahassee, FL, USA). CFU of *C. jejuni* and anti-IBDV-IgG-type antibody levels between groups were compared by using Two Sample T test or Wilcoxon Rank sum T test. The differences in immune cell populations and the cytokine expression pattern in the BF between multiple groups were analyzed by Kruskal–Wallis all pairwise comparison test. The differences in immune cell populations in the caecum, the anti-IBDV and anti-*C. jejuni* antibodies between multiple groups were evaluated by one-way-ANOVA-Tukey’s HSD test. Graphs were prepared with GraphPad v6 (Prism, LaJolla, USA). Differences are considered significant at *P* < 0.05.

## Results

### vvIBDV induced immunosuppression and effects of co-infection on viral pathogenesis

**Fig. 1:**
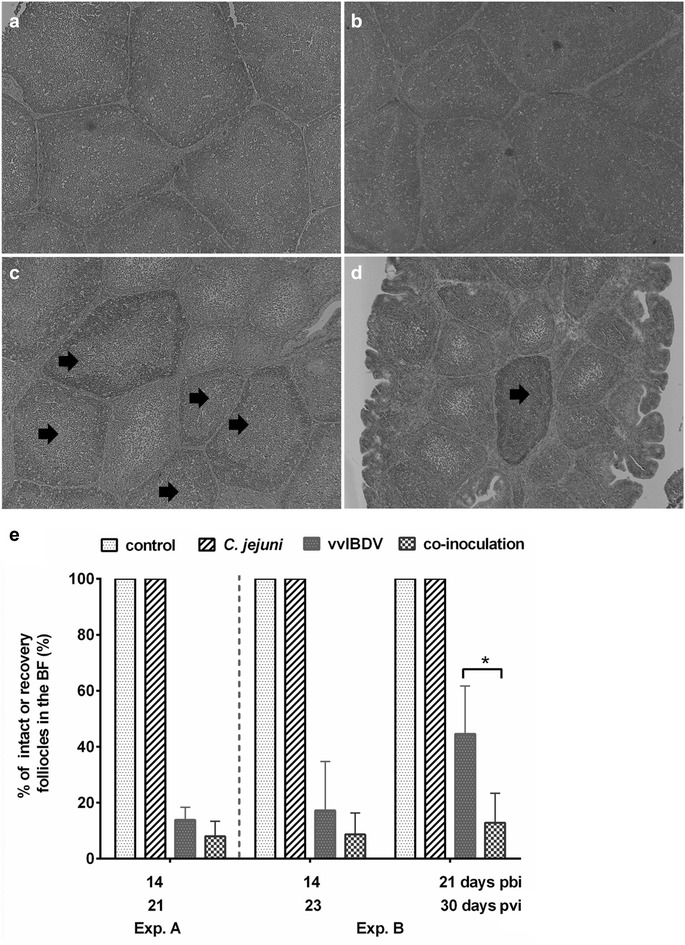
Histological evaluation of bursal recovery of chickens after vvIBDV-mono-inoculation and *C.jejuni* coinoculation (Exp. B as a representative experiment). Representative bursa sections of control (**a**), *C. jejuni*- (**b**), vvIBDV- (**b**), and vvIBDV + *C. jejuni* coinoculated (**d**) chickens at 21 days pbi. Arrows indicate recovering follicles B. **e** A summary of bursal recovery in the BF of chickens at different time points (Exp. A and Exp. B are separated by a dotted line) Error bar indicates the standard deviation (SD), ^*^indicates significant differences between the two indicated groups at the indicated time point (*P *< 0.05, n = 6/group). *pbi* post bacterial inoculation; *pvi* post IBDV (virus) inoculation

A similar pattern of anti-IBDV-maternally derived antibody (MDA) decline was observed in both experiments with ELISA-titers ranging from an average of log_10_ 3.7 ± 0.1 to 3.2 ± 0.4 in Exp. A and 3.7 ± 0.3 to 3.3 ± 0.3 in Exp. B at seven and 14 days post hatch, respectively. At 14 days post hatch, anti-IBDV-MDA titers were below the break-through level of vvIBDV [24]. Birds were randomly divided into two groups: one group was inoculated with vvIBDV and the other was inoculated with diluent PBS as control. vvIBDV induced a significant reduction in the number of circulating B cells in both experiments. Birds were inoculated with *C. jejuni* at 7 or 9 days pvi, when the percentage of circulating B cells was significantly reduced in comparison to non-inoculated groups in both experiments (Additional file 1: Figure S1, *P* < 0.05). At necropsy this was confirmed and vvIBDV had led to a significant bursal atrophy in virus-inoculated groups compared to the virus-free controls at the various necropsy days (data not shown). High IBDV-antigen-loads (S2 Fig) and severe histological lesions (Additional file 2: Table S1) were seen in the BF of all virus-infected groups. No significant differences were detected in the number of IBDV-antigen-positive cells neither between virus-inoculated and vvIBDV + *C. jejuni* co-inoculated groups nor between experiments (Additional file 3: Figure S2). Co-inoculation with *C. jejuni* did not significantly affect IBDV-lesion development up to 7 days pbi (14 and 16 days pvi in Exp. A and B, respectively), nor IBDV-antibody development (*P* > 0.05, data not shown). At the final day of each experiment, IBDV serum antibody levels in both vvIBDV mono-inoculated as well as vvIBDV + *C. jejuni* co-inoculated birds reached log_10_ titers of an average of 3.9 and 3.8 in Exp. A and B, respectively. Starting at 21 and 23 days pvi in Exp. A and B, respectively, there was a trend of a slower bursal recovery from histological lesions of co-inoculated groups compared to vvIBDV mono-inoculated ones, which was significant at 30 days pvi in Exp. B (Fig. 1).

### Effect of vvIBDV inoculation on *C. jejuni* colonization

**Fig. 2:**
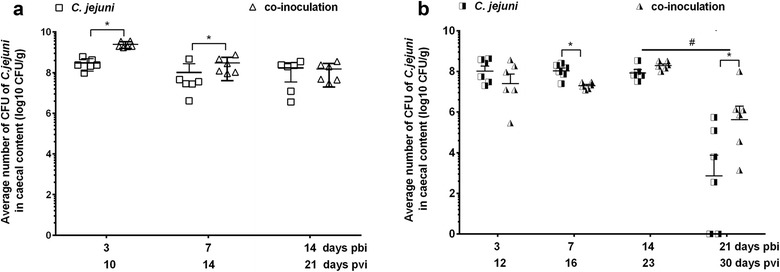
Average colony forming units (CFU) of *C. jejuni* in the caecal content of *C. jejuni*-mono-inoculated and co-inoculated birds of Exp. A (**a**) and Exp. B (**b**). *pbi* post bacterial inoculation; *pvi* post IBDV (virus) inoculation. *C. jejuni *= *C. jejuni* mono-inoculated group, co-inoculation = vvIBDV + *C. jejuni* co-inoculated group. Error bar indicates the standard deviation (SD). *indicates significant difference between two groups at the indicated days pbi (*P < 0.05, n = 6/group)

All groups were *C. jejuni*-negative at the day of bacterial inoculation and all the non-*C. jejuni*-inoculated groups remained *C. jejuni*- free throughout both experiments. To investigate the effect of vvIBDV inoculation on the *C. jejuni* colonization pattern, two different time intervals between the inoculation of virus and bacteria were selected. Overall, comparable numbers of *C. jejuni* CFU were detected in mono-inoculated groups between experiments with an average of log_10_ of 8.0 ± 0.6, 8.0 ± 0.4, 7.9 ± 0.4 CFU at 3, 7, and 14 days pbi, respectively (Fig. 2). In Exp. A, significantly higher CFU numbers were observed in the caecal content of co-inoculated birds compared to *C. jejuni* mono-inoculated ones at 3 and 7 days pbi (Fig. 2a, *P *< 0.05). In Exp. B. CFU were significantly lower by 5.4 fold in the co-inoculated birds at seven days pbi compared to *C. jejuni* mono-inoculated ones (Fig. 2b, *P *< 0.05). While *C. jejuni* mono-inoculated birds showed a significant bacterial clearance at 21 days pbi, with two birds being negative for *C. jejuni* in caecal content and four birds with CFU of log_10_ of 4.3 ± 1.3, co-inoculated birds were all still colonized with *C. jejuni* with CFU of a range of log_10_ of 5.6 ± 1.6. Reisolation attempts of *C. jejuni* from ileum confirmed clearance of *C. jejuni,* in 50% of mono-inoculated birds compared to 100% *C. jejuni*-positive co-inoculated birds at 21 days pbi (Additional file 4: Table S2).

vvIBDV-inoculation did not substantially affect the dissemination of *C. jejuni* to different extraintestinal organs (Additional file 4: Table S2). Co-inoculated birds had a higher percentage of *C. jejuni*-positive birds (83%) in comparison to *C. jejuni* mono-inoculated birds (33%) at 21 days pbi in the bursa of Fabricius (Additional file 4: Table S2).

### Effect of vvIBDV on *C. jejuni* induced lesion development

Neither clinical signs such as depression or diarrhea nor mortality were observed in any of the groups during both experiments. No significant difference in body weight development was observed between the four groups during Exp. A and B (data not shown, *P* > 0.05). Histological investigations of caecum sections did not reveal significant lesions in any of the investigated groups. vvIBDV-inoculation led in all groups to a slight infiltration of heterophils in the lamina propria as well as the submucosal area (data not shown), which was not observed in the virus-free groups.

### Effect of vvIBDV-*C. jejuni* co-inoculation on T and B lymphocytes in the BF and LP of the caecum

Both vvIBDV and *C. jejuni* influenced the local immune response. Consistent with previous studies [26], vvIBDV inoculation led to an increase in T lymphocytes (CD4 + and CD8ß +), as well as a depletion of B lymphocytes in the BF in the vvIBDV mono-inoculated birds (data not shown). *C. jejuni* had no significant effect on lymphoid cell populations in the BF (*P* > 0.05), and in co-inoculated birds, numbers were comparable to vvIBDV mono-inoculated chickens (data not shown).

**Fig. 3:**
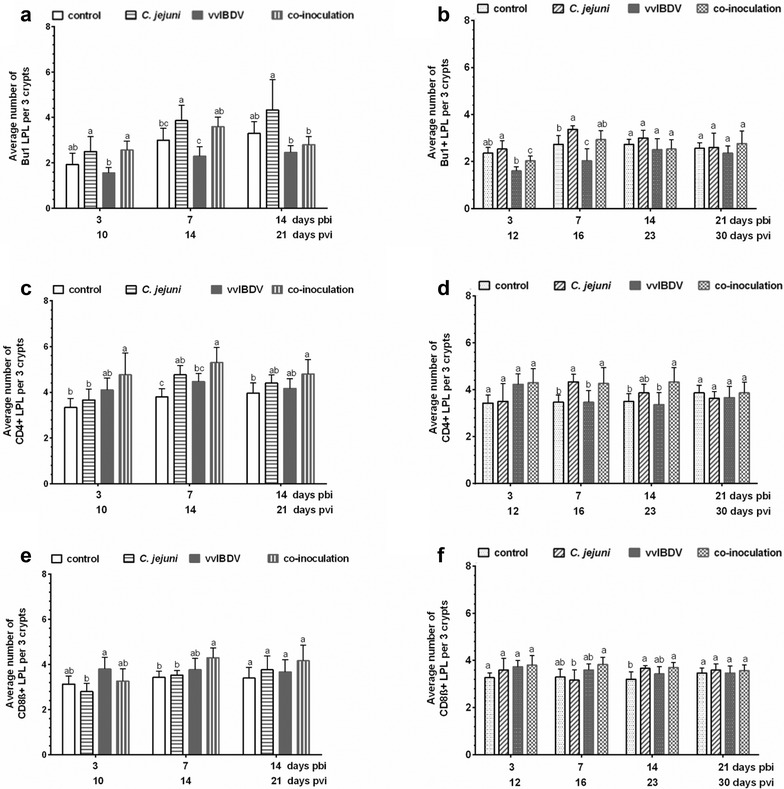
Immunohistochemical detection of Bu1+ B (**a**, **b**) and CD4+ (**c**, **d**) as well as CD8 + (**e**, **f**) T cells in the lamina propria of the caecum of mono- and co-inoculated birds of Exp. A (**a**, **c**, **e**) and Exp. B (**b**, **d**, **f**) (n = 6/group). *pbi* post bacterial inoculation; *pvi* post IBDV (virus) inoculation. Error bars indicate the standard deviation (SD). ^abc^letters indicate significant differences between groups within the same experiment at the indicated time points (*P *< 0.05). control = non-inoculated control, *C. jejuni *= *C. jejuni* mono-inoculated group, vvIBDV = vvIBDV mono-inoculated group, co-inoculation = vvIBDV + *C. jejuni* co-inoculated group

In the caecum, vvIBDV mono-inoculation led to a decrease in the number of LP B lymphocytes (LPL), which was significant at 7 days pbi in both experiments (Fig. 3a and b, *P *< 0.05, T test). vvIBDV also induced an increase of CD4+ and CD8ß+ T LPL at 10–12 pbi compared to the control groups, which neither received vvIBDV nor *C. jejuni*, (Fig. 3c and d). Also *C. jejuni*-mono-inoculated groups showed increased numbers of T and also B cells compared to non-inoculated controls at some investigated time points as published before [3, 18], which was significant for B and CD4+ T cells at seven days pbi in both experiments (*P* < 0.05, T test for the comparison of *C. jejuni*-mono-inoculated and control groups), and for CD8+ T cells at 14 days pbi in the Exp. B (*P* < 0.05, T test for the comparison of *C. jejuni*-mono-inoculated and control groups).

In co-inoculated birds an increase in the number of LP B lymphocytes was observed at 7 days pbi compared to non-inoculated controls, which was comparable to the *C. jejuni*-mono-inoculated group in both experiments (Fig. 3a, b, *P *< 0.05). This was followed by a decrease going in line with the vvIBDV-mono-inoculated chickens, but this difference was not significant compared to the non-inoculated controls (Fig. 3a, b, *P* > 0.05). An increase in the number of T LPL was observed in the co-inoculated birds compared to the non-inoculated controls at most investigated time points pbi both in Exp. A and B (Fig. 3c–f). Numbers were comparable at most time points to *C. jejuni* mono-inoculated groups (Fig. 3, *P *< 0.05).

### Effect of vvIBDV-*C. jejuni* co-inoculation on cytokine expression

**Fig. 4:**
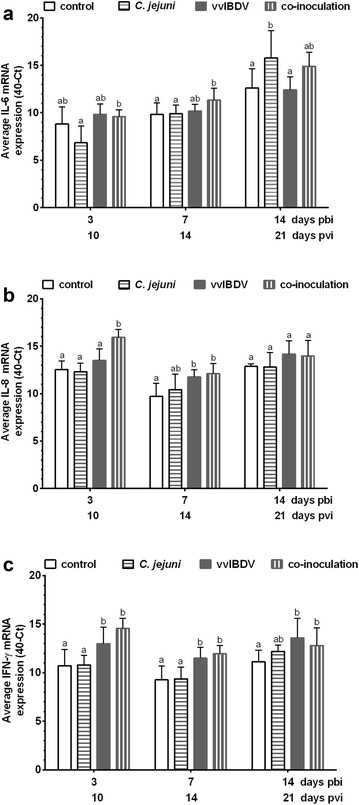
mRNA expression of IL-6 (**a**), IL-8-homolog (**b**) and IFN-γ (**c**) in the BF of birds of Exp. A (n = 6/group). The data is presented as 40-CT normalized to 28S. *pbi* post bacterial (*C. jejuni*) inoculation; *pvi* post IBDV (virus) inoculation. Error bars indicate the standard deviation (SD), ^abc^letters indicate significant differences between groups within the same experiment at the indicated time points (*P *< 0.05). control = non-inoculated control, *C. jejuni *= *C. jejuni* mono-inoculated group, vvIBDV = vvIBDV mono-inoculated group, co-inoculation = vvIBDV + *C. jejuni* co-inoculated group

vvIBDV mono-inoculation significantly affected the mRNA expression of IL-8 and IFN-γ in the BF. In Exp. A a significant upregulation of IL-8 was detected at 7 days pvi compared to the non-inoculated controls (*P* < 0.05). A significant upregulation of IFN-γ was observed in Exp. A at all investigated time points in the vvIBDV mono-inoculated birds in comparison to non-inoculated controls (Fig. 4, *P *< 0.05), which was confirmed for day 3 pbi in Exp. B (the only investigated time point in this experiment, data not shown).

*Campylobacter jejuni* inoculation led to a significant upregulation of IL-6 at 14 days pbi in the BF compared to non-inoculated controls (Exp. A, Fig. 4a, *P *< 0.05), while the other two cytokines were not affected.

Co-inoculation led to a significant upregulation of IL-6 at 7 days pbi compared to non-inoculated controls in Exp. A. An upregulation of IL-8 gene expressions in co-inoculated birds was observed at 3 and 7 days pbi compared to non-inoculated controls, and a significant upregulation of IFN-γ was detected at all the investigated time points in the co-inoculated birds in comparison to non-inoculated controls (Fig. 4, *P *< 0.05). There were no differences in cytokine expression pattern between co-inoculated and vvIBDV mono-inoculated birds at most investigated time points (*P* > 0.05). The changes in cytokine expression in co-inoculated birds at 3 days pbi were also confirmed in Exp. B (data not shown).

### Effect of vvIBDV-*C. jejuni* coinoculation on *C. jejuni*-antibody development

**Fig. 5:**
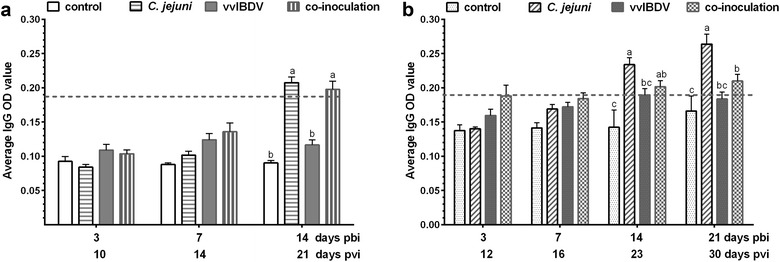
Induction of *C. jejuni*-specific IgG-type antibodies in serum of mono- and co-inoculated birds of the Exp. A (**a**) and Exp. B (**b**). pbi = post *C. jejuni* bacterial inoculation; pvi = post IBDV (virus) inoculation. Error bars indicate the standard deviation (SD), ^abc^letters indicate significant differences between groups within the same experiment at the indicated time points (*P *< 0.05). control = non-inoculated control, *C. jejuni *= *C. jejuni* mono-inoculated group, vvIBDV = vvIBDV mono-inoculated group, co-inoculation = vvIBDV + *C. jejuni* co-inoculated group. Dotted line shows the cut-off level

Consistent with previous studies [3, 18] a significant upregulation of *C. jejuni*-specific antibodies was observed starting at 14 days pbi in the serum of *C. jejuni* mono-inoculated and co-inoculated birds compared to non-*C. jejuni*-inoculated controls in both experiments (*P *< 0.05), there was a trend that antibody levels were lower in the co-inoculated group. At 21 days pbi significantly lower levels of IgG *C. jejuni*-specific antibodies were detected in co-inoculated birds in comparison to *C. jejuni* mono-inoculated ones (Fig. 5b, *P *< 0.05).

### Effect of vvIBDV-*C. jejuni* co-inoculation on the gut microbiota

**Fig. 6:**
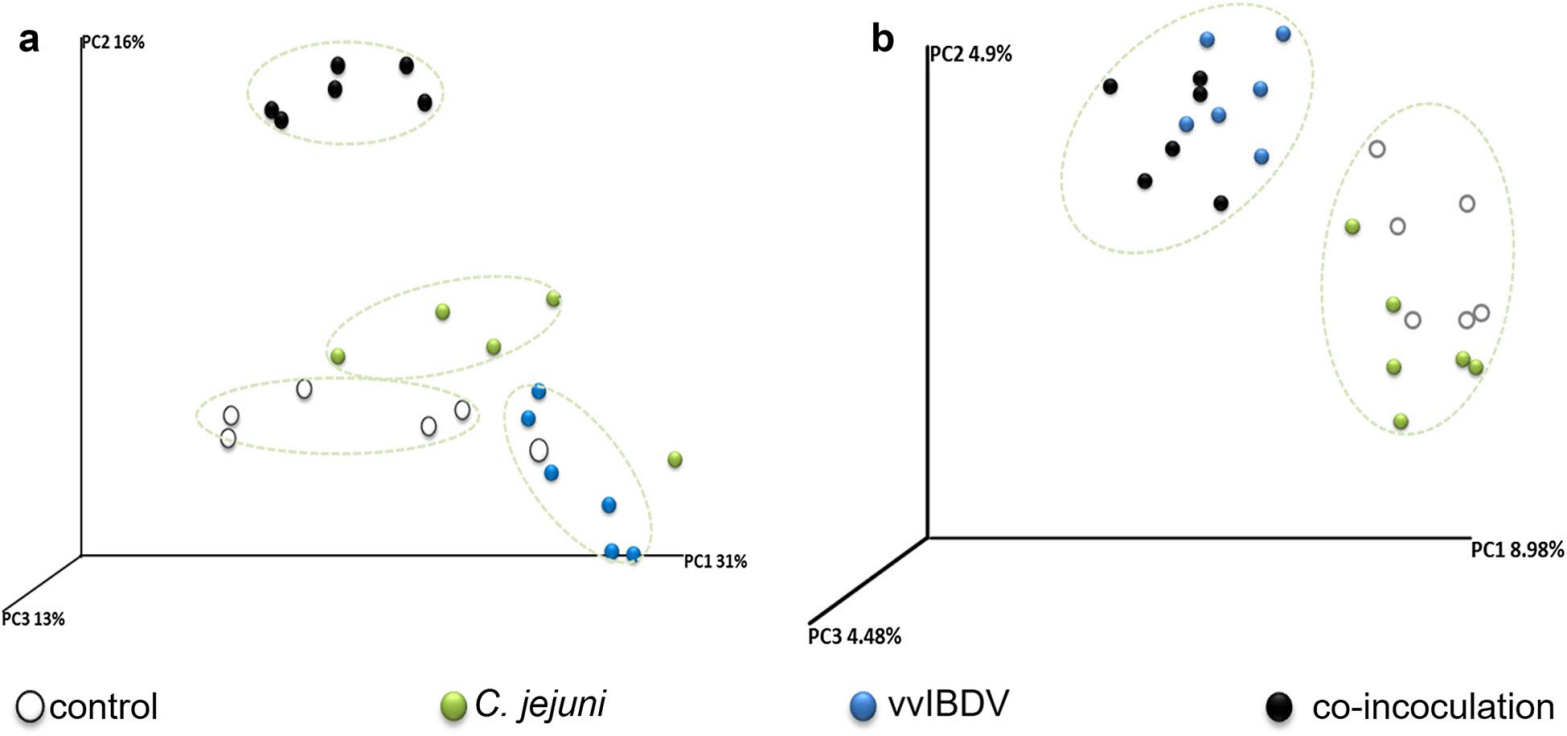
Microbiota diversity in the caecal content of birds from the Exp. A (**a**) (14 days pvi) and Exp. B (**b**) (16 days pvi) at 7 days pbi. UniFrac analysis followed by PCoA indicates variability in the caecal microbiota composition of birds. Control = non-inoculated control, *C. jejuni *= *C. jejuni* mono-inoculated group, vvIBDV = vvIBDV mono-inoculated group, co-inoculation = vvIBDV + *C. jejuni* co-inoculated group. pbi = post bacterial (*C. jejuni*) inoculation

UniFrac analysis followed by PCoA using weighted and unweighted analysis indicated mono-inoculations had some effects on the gut microbiota in both experiments at 7 days pbi. vvIBDV mono-inoculated birds showed a clear separation from non-inoculated controls (Fig. 6a and b). *C. jejuni* mono-inoculated groups showed a separation from the non-inoculated controls in Exp. A but not in Exp. B. pbi = post bacterial (*C. jejuni*) inoculation.

The effect of co-inoculation on gut microbiota was different between Exp. A and Exp. B. In Exp. A, gut microbiota composition was significantly modified due to the co-inoculation, and clearly different to all other three groups. In Exp. B, co-inoculated and vvIBDV-inoculated birds grouped together and were clearly separated from the *C. jejuni* mono-inoculated and non-inoculated control groups. The abundance of *Campylobacter* significantly increased in the co-inoculated birds (2.76%) compared to *C. jejuni* mono-inoculated ones (2.40%) in Exp. A. The opposite was observed for Exp. B (co-inoculated birds (8.83%), *C. jejuni* mono-inoculated (10.72%)), which confirmed the data of the CFU in the caecal content at this time point. In both experiments, we observed that *C. jejuni* inoculation induced a significant lower abundance of *Lachnospiracea incertae sedis* and *Roseburia* in both experiments, and a lower abundance of *Eubacterium* in the caecal content of *C. jejuni* mono-inoculated birds compared to non-inoculated controls, which was significant in Exp. B (Table 1). In addition vvIBDV infection led to a slight difference in the abundance of *Faecalibacterium* between Exp. A and Exp. B compared to control. A significant higher abundance of *Faecalibacterium* was observed in vvIBDV-mono-inoculated birds compared to non-inoculated controls in Exp. A, while in Exp. B only a significant lower abundance was detected (Table 1, *P* < 0.05). Co-inoculated birds showed a significant lower abundance of *Lachnospiracea incertae sedis* in the caecum compared to non-inoculated controls in both experiments (Table 1).

**Table 1:** Analysis of microbiota composition in mono-and co-inoculated groups in comparison to non-inoculated controls at 7 days pbi

Genus	Significant differences as indicated by * in the abundance of bacterial genus in					
	Exp. A			Exp. B		
	*C. jejuni*	vvIBDV	Co-inoculation	*C. jejuni*	vvIBDV	Co-inoculation
*Campylobacter*	*	–	*	*	–	*
*Clostridium XlVa*	*	*	*	*	*	–
*Eubacterium*	–	–	*	*	*	*
*Faecalibacterium*	–	*	–	–	*	–
*Lachnospiracea incertae sedis*	*	*	*	*	–	*
*Lactobacillus*	*	–	–	*	–	–
*Roseburia*	*	*	*	–	–	*

* Significant differences in the abundance of OTUs, *P* < 0.05 (2-sample T test)

–: no significant differences (*P* > 0.05); pbi: post bacterial (*C. jejuni*) inoculation

## Discussion

In this study we investigated the effect of vvIBDV-induced immunosuppression on *C. jejuni* colonization. Two experiments were conducted using broiler chicks from the same genotype with comparable IBDV-maternally derived antibody levels.Broiler chickens were inoculated with vvIBDV at 14 days post hatch. Virus replication, as indicated by high numbers of IBDV-antigen positive cells in the BF, was confirmed [6]. Monitoring of circulating B cells showed a significant decrease in relative B cell numbers from 5 days pvi onwards (*P *< 0.05) compared to the virus-free controls. We also observed bursal atrophy with B cell depletion. vvIBDV-antigen-loads, sero-conversion as well as bursa lesion development were comparable between Exp. A and B. These results are consistent with previous studies confirming vvIBDV infection and immunosuppression [5, 7, 8]. Although previous studies indicated an effect of IBDV on *C. jejuni*-colonization (Subler et al. [11]; Stojanov et al. [12]), the influence of the time interval between the inoculations of the pathogens on the outcome of infections was not investigated. Co-inoculation experiments with various other pathogens in vitro and in vivo demonstrated clearly a significant influence of the time interval on the pathogenesis of either infectious agent [27, 28]. Two different time points after vvIBDV-inoculation were selected for *C. jejuni*-inoculation. Seven and 9 days pvi were chosen, which was after the acute phase of vvIBDV infection, when the effects on B cells were expected to be most prominent and numbers of B cells were clearly depressed but the vvIBDV-induced cytokine storm may have waned [5, 20]. It would have been desirable to investigate both time points of *C. jejuni*-inoculation within one experiment, which was not possible in this experiment due to logistic constrains. But the comparable outcome of vvIBDV-infection and the comparable *C. jejuni* colonization rate between three and 14 days pbi in mono-inoculated groups in both experiments allows the direct comparison of both experiments.

Interestingly, co-inoculation with *C. jejuni* may delay follicular recovery, although the mechanisms behind this observation that are not clear.

Two days difference in *C. jejuni*-inoculation after vvIBDV-infection had a significant effect on the outcome of *C. jejuni* colonization. In Exp. A the early phase of *C. jejuni* colonization was clearly impacted by vvIBDV, leading to 2.8- to 8.1-fold higher CFU in co-inoculated birds compared to *C. jejuni* mono-inoculated ones. In Exp. B vvIBDV affected mainly the colonization pattern at 21 days pbi, which might correlate with the clearance of *C. jejuni*. Co-inoculated birds had significant higher CFU with all six birds having a CFU range between log_10_ of 3.2 and 8 in the caecum compared to the mono-inoculated group, in which two birds were *C. jejuni* negative by culture of caecal content and four had CFU of 4.3 ± 1.7 at 21 days pbi (*P* < 0.05). Possibly this effect would also have been seen in Exp. A, if the experiment would have been extended. Interestingly this significantly higher bacterial load in co-inoculated birds at 21 days pbi coincided with a reduced *C. jejuni*-specific antibody response compared to the *C. jejuni* mono-inoculated groups (*P* < 0.05). Antibody levels were significantly lower at 21 days pbi in co-inoculated birds compared to *C. jejuni* mono-inoculated ones in Exp. B. A trend of a compromised anti-*C. jejuni*-antibody response was already visible at 14 days pbi in both experiments. These results provide circumstantial evidence that *C. jejuni*-infection may be controlled by humoral immunity as suggested before [14]. In Cyclophosphamide-immunocompromised birds the same trend was observed that antibodies may play a significant role in the bacterial clearance phase later after inoculation [16]. Further studies need to be done, to evaluate the local *C. jejuni*-specific IgA-response to confirm the role of local humoral immunity in *C. jejuni*-colonization.

An additional confirmation of vvIBDV-induced B cell depletion, and the lack or reduced stimulation of the B cell-mediated anti-*C. jejuni*-response is provided by the reduced number of LP B lymphocytes in co-inoculated birds compared to *C. jejuni* mono-inoculated ones (*P* < 0.05). While *C. jejuni* mono-inoculation led to an upregulation of LP B lymphocytes starting at three days pbi in Exp. A, and on days three and seven pbi in Exp. B, there was only a detectable B cell upregulation in the co-inoculated groups at seven days pbi in both experiments. At 14 days pbi local B cell numbers were reduced in co-inoculated groups compared to most other groups.

This effect of vvIBDV on the *C. jejuni*-specific antibody response is interesting, as this effect was most prominent at 21–23 days pvi, when virus clearance in the BF was very much advanced and beginning bursal recovery was observed in both vvIBDV-inoculated groups. This clearly shows that B cell immunosuppression lasts beyond the acute phase and the effects on secondary infections may be detectable over 14 days post vvIBDV-infection. Previous studies demonstrated that bursal recovery with two different types of follicle structures starts 5 weeks post IBDV infection [8, 29, 30]. The larger follicles are correlated with partial recovery of the antibody responsiveness, while the smaller follicles appear unable to produce antigen-responsive B cells [29, 30], which suggested that immunosuppression was maintained. In Exp. A other immunosuppressive or immunomodulatory effects beside the depression of the humoral immunity may have additionally affected *C. jejuni* colonization as already early bacterial replication was exacerbated in co-inoculated groups at 3 and 7 days pbi. It was shown in a variety of other studies, that during the early phase of IBDV infection not only the humoral immunity may be compromised in IBDV-infected birds but also macrophage activity, and indirectly the cell-mediated immunity [5, 7].

There was no information available regarding the effect of vvIBDV infection on the gut microbiota in chickens. It may be speculated that due to its immunosuppressive effects on the gut-associated immune system [13] IBDV may indirectly modulate the composition of the microflora. Effects like that were previously seen in chickens infected with Marek’s disease virus (MDV) [31], and also in humans after infection with human immunodeficiency virus (HIV) [32]. Interestingly, vvIBDV modified the gut-microbiota composition in both experiments compared to control birds as indicated by the PCoA analysis (Fig. 6). While in Exp. A, there was a clear separation between all groups in the composition of the microbiota composition, in Exp. B, non-inoculated controls and *C. jejuni*-mono-inoculated chickens grouped together as well as the vvIBDV-infected and co-inoculated ones. This suggests that at least in Exp. B vvIBDV-effects on the microbiota dominate also in co-inoculated groups, which is interesting and the meaning for the host has to be investigated further. We observed that *C. jejuni*-infected birds had a higher abundance of *Clostridiaceae* in the caecal content in both experiments, which confirms previous studies [3, 33]. *C*. *jejuni* colonization results in hydrogen consumption, which promotes the growth of some *Clostridium* sp. through increased fermentation, leading to an increased organic acid production, which subsequently allows *C*. *jejuni* to use organic acids as an energy source [34]. *Faecalibacterium*, a butyrate producer, dominates in the caecal microbiota of chickens at approximately 3 weeks of age [35]. High numbers of *Faecalibacterium* may be detrimental for *C*. *jejuni* since butyrate may inhibit replication of *C*. *jejuni* [36]. Different colonization rates between *C*. *jejuni* mono-inoculated and co-inoculated birds might have been due to the different abundance of *Faecalibacterium* in the gut microbiota. In Exp. B, a higher abundance of *Faecalibacterium* (13.95%) was correlated with a lower abundance of *Campylobacter* (8.83%) in the *C. jejuni* mono-inoculated birds compared to co-inoculated birds, which showed a lower abundance of *Faecalibacterium* (11.07%) with a higher abundance of *C. jejuni* (10.72%). Further studies on the interaction of the gut microbiota and *C. jejuni* need to be conducted to elucidate the exact mechanisms being involved in the control of *C. jejuni* in the gut. This may subsequently help to develop better control strategies and ultimately reduce the colonization rate and therefore the risk for infections of humans after chicken meat consumption [37].

The role of T cells in the control of *C. jejuni* was demonstrated in mice and humans, but little is known about T cell responses in chickens. In our study a significant up-regulation of T lymphocytes was observed locally in the caecum of *C. jejuni* mono-inoculated and co-inoculated birds compared to control birds. This observation coincides with previous studies suggesting that the presence of these CD4^+^ and CD8^+^ LP T lymphocytes is due to an intraluminal antigenic stimulus [3]. It was suggested that activated T cells may play a protective role in the host defense against *C. jejuni* infection [3, 38]. Especially regulatory T cells (Tregs) may be import in the regulation of inflammatory versus anti-inflammatory conditions in the gut [39, 40]. vvIBDV did not affect this *C. jejuni*-mediated T cell response in the gut. Co-infected groups showed a comparable number of LP T lymphocytes as *C. jejuni*-mono-inoculated ones, which did not correlate with the increased number of CFU in these groups suggesting a neglectable role of these cells in the control of *C. jejuni* under our experimental conditions.

Cytokine expression pattern were either influenced by *C. jejuni* or vvIBDV. Co-inoculations only had an enhancing effect on IL-8 mRNA expression at 3 days pbi compared to the other groups, but the consequences for the pathogenesis are not known. The expression pattern of IL-6 is dominated by vvIBDV during the acute phase of *C. jejuni*-colonization, and later on by *C. jejuni* suggesting that this cytokine may not play a key role in the early control of *C. jejuni.*

## Conclusion

Our findings demonstrated a significant influence of vvIBDV infection on different immune reactions, microbiota composition, and subsequently on the colonization pattern *C. jejuni*. Our data suggests an important role of the humoral immunity especially in the clearance of *C. jejuni*. Further studies should address experimental days beyond 21 days pbi, in order to investigate this influence of humoral immunity on *C. jejuni* further. Other immune mechanisms being involved in the control of *C. jejuni* may also be affected by vvIBDV, which may explain the enhancement of CFU in co-inoculated birds in Exp. A during the early phase after bacterial inoculation. The role of the cell-mediated immunity, especially Tregs, is less clear, and has to be investigated further, but to address this vvIBDV-infection may not be a suitable model.

## Additional files

**Additional file 1: Figure S1.** Flow cytometric analysis of Bu1+ B cells in peripheral blood lymphocytes (PBL) of broiler chickens at different days pvi from Exp. A (A) and Exp. B (B). *indicates significant differences between non-inoculated control and vvIBDV inoculated birds (*P* < 0.05). Error bars indicate standard deviation. Control = virus-free control, vvIBDV = vvIBDV-inoculated group, pvi = post IBDV inoculation.

**Additional file 2: Table S1.** Histological bursal lesion of chickens at different time points after vvIBDV and *C. jejuni*-inoculation. ND = not done; SD = standard deviation; Exp. = experiment; BF = bursa of Fabricius; pbi = post bacterial (*C. jejuni*) inoculation. Different superscript letters indicate significant differences between groups (*P* < 0.05).

**Additional file 3: Figure S2.** Immunohistochemical detection of IBDV antigen in the BF of chickens from Exp. A (A) and Exp. B (B). vvIBDV = vvIBDV mono-inoculated group, co-inoculation = vvIBDV + *C. jejuni* co-inoculated group.

**Additional file 4: Table S2.** Detection of *C. jejuni* presence in different organs at different days pbi. Swabs samples of bursa, spleen and ileum were collected and investigated for *C. jejuni* presence by plating on CCDA plates. The livers were collected, homogenized and investigated by plating on CCDA plates. Non-inoculated groups remained *C. jejuni* negative throughout the experiments, pbi =  post bacterial (*C. jejuni*) inoculation. *C. jejuni =* *C. jejuni* mono-inoculated group, co-inoculation=vvIBDV + *C. jejuni* co-inoculated group.

## Abbreviations

*C. jejuni*: *Campylobacter jejuni*
BF: bursa of Fabricius
CCDA: charcoal cefoperazone deoxycholate agar
CFU: colony forming units
EID: egg infectious dose
ELISA: enzyme-linked immunosorbent assay
Exp.: experiment
IBDV: infectious bursal disease virus
IL: interleukin
LP: lamina propria
LPL: lamina propria lymphocyte
MDA: maternally derived antibody
OTU: operational taxonomic units
pbi: post bacteria inoculation
PBS: phosphate buffered saline
PCoA: principal coordinate analysis
pvi: post IBDV (virus) inoculation
vvIBDV: very virulent IBDV
FBS: fetal bovine serum
CMI: cell-mediated immune responses
