# Supplementary material for: Infectious bursal disease virus inoculation infection modifies Campylobacter jejuni–host interaction in broilers
Source: Gut Pathog. 2018 Mar 30;10:13. doi: 10.1186/s13099-018-0241-1 (PMC5877392; doi:10.1186/s13099-018-0241-1)
Supplement: Supplementary file 2 — Additional file 2: Table S1. Histological bursal lesion of chickens at different time points after vvIBDV and C. jejuni-inoculation. ND = not done; SD = standard deviation; Exp. = experiment; BF = bursa of Fabricius; pbi = post bacterial (C. jejuni) inoculation. Different superscript letters indicate significant differences between groups (P < 0.05). [file 13099_2018_241_MOESM2_ESM.docx]

**Table S1. Histological bursal lesions of chickens at different time points after vvIBDV and *C. jejuni*-inoculation.**

| Exp. | vvIBDV | *C.jejuni* | Percentage of bursal follicles with lymphoid cell depletion in the BF ± SD (%) on days pbi | | | |
| --- | --- | --- | --- | --- | --- | --- |
|  |  |  | 3 | 7 | 14 | 21 |
| Exp. A | **-** | **-** | 0 ± 0^a^ | 0 ± 0^a^ | 0 ± 0^a^ | ND |
|  | **-** | **+** | 0 ± 0^a^ | 0 ± 0^a^ | 0 ± 0^a^ | ND |
|  | **+** | **-** | 93 ± 4^b^ | 82 ± 9^b^ | 71 ± 17^b^ | ND |
|  | **+** | **+** | 87± 8^b^ | 89 ± 8^b^ | 78 ± 7^b^ | ND |
|  | | | | | | |
| Exp. B | **-** | **-** | 0 ± 0^a^ | 0 ± 0^a^ | 0 ± 0^a^ | 0 ± 0^a^ |
|  | **-** | **+** | 0 ± 0^a^ | 0 ± 0^a^ | 0 ± 0^a^ | 0 ± 0^a^ |
|  | **+** | **-** | 92 ± 5^b^ | 64 ± 10^b^ | 63±10^b^ | 59 ± 17^b^ |
|  | **+** | **+** | 90 ± 8^b^ | 74 ± 18^b^ | 78±7^b^ | 64 ± 15^b^ |

ND=not done; SD=standard deviation; Exp.=experiment; BF=bursa of Fabricius. pbi=post bacterial (*C. jejuni*) inoculation. Different superscript letters indicate significant differences between groups (*P* < 0.05).
