# Supplementary material for: Infectious bursal disease virus inoculation infection modifies Campylobacter jejuni–host interaction in broilers
Source: Gut Pathog. 2018 Mar 30;10:13. doi: 10.1186/s13099-018-0241-1 (PMC5877392; doi:10.1186/s13099-018-0241-1)
Supplement: Supplementary file 4 — Additional file 4: Table S2. Detection of C. jejuni presence in different organs at different days pbi. Swabs samples of bursa, spleen and ileum were collected and investigated for C. jejuni presence by plating on CCDA plates. The livers were collected, homogenized and investigated by plating on CCDA plates. Non-inoculated groups remained C. jejuni negative throughout the experiments, pbi = post bacterial (C. jejuni) inoculation. C. jejuni = C. jejuni mono-inoculated group, co-inoculation=vvIBDV + C. jejuni co-inoculated group. [file 13099_2018_241_MOESM4_ESM.docx]

**Table S2. Detection of *C. jejuni* presence in different organs at different days pbi**

|  | Sample sites |  | % of *C.jejuni-*positive birds at different days pbi | | | | | | | |
| --- | --- | --- | --- | --- | --- | --- | --- | --- | --- | --- |
|  |  |  | 3 | | 7 | | 14 | | 21 | |
|  |  |  | *C. jejuni* | Co-inoculation | *C. jejuni* | Co-inoculation | *C. jejuni* | Co-inoculation | *C. jejuni* | Co-inoculation |
| Exp.A | spleen |  | 33 | 100 | 17 | 17 | 17 | 33 | ND | ND |
|  | liver homogenate |  | 67 | 100 | 100 | 100 | 100 | 100 | ND | ND |
|  | bursa |  | 83 | 100 | 100 | 100 | 83.3 | 100 | ND | ND |
|  | ileum |  | 100 | 100 | 100 | 100 | 100 | 100 | ND | ND |
|  |  |  |  |  |  |  |  |  |  |  |
| Exp.B | spleen |  | 0.0 | 0.0 | 83 | 33 | 40.0 | 17 | 50 | 67 |
|  | liver homogenate |  | 50 | 50 | 100 | 100 | 100 | 33 | 17 | 17 |
|  | bursa |  | 67 | 100 | 100 | 100 | 100 | 83 | 33 | 83 |
|  | ileum |  | 100 | 100 | 100 | 100 | 100 | 100 | 50 | 100 |

Swabs samples of bursa, spleen and ileum were collected and investigated for *C. jejuni* presence by plating on CCDA plates. The livers were collected, homogenized and investigated by plating on CCDA plates. Non-inoculated groups remained *C. jejuni* negative throughout the experiments. pbi=post bacterial (*C. jejuni*) inoculation. *C. jejuni=C. jejuni* mono-inoculated group, co-inoculation=vvIBDV+*C. jejuni* co-inoculated group.
